# Fetal-Adult Cardiac Transcriptome Analysis in Rats with Contrasting Left Ventricular Mass Reveals New Candidates for Cardiac Hypertrophy

**DOI:** 10.1371/journal.pone.0116807

**Published:** 2015-02-03

**Authors:** Katja Grabowski, Mona Riemenschneider, Leonard Schulte, Anika Witten, Angela Schulz, Monika Stoll, Reinhold Kreutz

**Affiliations:** 1 Department of Clinical Pharmacology and Toxicology, Charité Centrum für Therapieforschung, Charité—Universitätsmedizin Berlin, Berlin, Germany; 2 Genetic Epidemiology, University of Münster, Münster, Germany; University Medical Center Utrecht, NETHERLANDS

## Abstract

Reactivation of fetal gene expression patterns has been implicated in common cardiac diseases in adult life including left ventricular (LV) hypertrophy (LVH) in arterial hypertension. Thus, increased wall stress and neurohumoral activation are discussed to induce the return to expression of fetal genes after birth in LVH. We therefore aimed to identify novel potential candidates for LVH by analyzing fetal-adult cardiac gene expression in a genetic rat model of hypertension, i.e. the stroke-prone spontaneously hypertensive rat (SHRSP). To this end we performed genome-wide transcriptome analysis in SHRSP to identify differences in expression patterns between day 20 of fetal development (E20) and adult animals in week 14 in comparison to a normotensive rat strain with contrasting low LV mass, i.e. Fischer (F344). 15232 probes were detected as expressed in LV tissue obtained from rats at E20 and week 14 (p < 0.05) and subsequently screened for differential expression. We identified 24 genes with SHRSP specific up-regulation and 21 genes with down-regulation as compared to F344. Further bioinformatic analysis presented Efcab6 as a new candidate for LVH that showed only in the hypertensive SHRSP rat differential expression during development (logFC = 2.41, p < 0.001) and was significantly higher expressed in adult SHRSP rats compared with adult F344 (+ 76%) and adult normotensive Wistar-Kyoto rats (+ 82%). Thus, it represents an interesting new target for further functional analyses and the elucidation of mechanisms leading to LVH. Here we report a new approach to identify candidate genes for cardiac hypertrophy by combining the analysis of gene expression differences between strains with a contrasting cardiac phenotype with a comparison of fetal-adult cardiac expression patterns.

## Introduction

Left ventricular (LV) hypertrophy (LVH) is a major sign of cardiac adaption to high blood pressure and the most common cause of cardiac hypertrophy [1]. Due to the high prevalence of arterial hypertension, LVH will soon become the most common risk factor for cardiac failure worldwide [2].

However, independent of blood pressure, LVH alone profoundly affects morbidity and mortality from cardiovascular diseases [3–5]. It is still poorly understood why only one part of hypertensive patients develop LVH over time. Family and population studies demonstrated that LV mass variation is clearly influenced by genetic factors [6,7]. This is also supported by genetic analysis of inbred rat models, including hypertensive rat strains [8,9].

Reactivation of fetal gene expression patterns has been demonstrated to play a crucial role in common cardiac diseases in adult life including LVH [10]. Thus, increased wall stress and neurohumoral activation are discussed to induce the return to expression of fetal genes after birth in LVH [11]. The switch from adult α-myosin heavy chain (MHC) isoform to fetal β-MHC isoform is a well-known process during cardiac hypertrophy [12]. Reinducing glucose metabolism under low oxygen supply in hypertrophied hearts is a further example for cardiac adaption processes [13,14].

In the presented work we aimed to test whether fetal gene expression programs are linked to the genetic predisposition to LVH. We performed genome-wide gene expression analysis in a genetic rat model of LVH, i.e. the stroke-prone spontaneously hypertensive rat (SHRSP), and in the normotensive rat strain Fischer (F344). SHRSP is a well-established model of polygenetic hypertension and hypertensive organ damage, including LVH. F344 was chosen as an inbred normotensive contrasting rat strain to SHRSP, because F344 exhibit a significantly lower cardiac mass compared to the historical comparator, i.e. Wistar-Kyoto rats (WKY) [15] and might be therefore more informative in comparison to SHRSP than WKY. In addition, F344 is recommended by the National Institute on Aging and the National Centre for Toxicological Research in the US as a reference strain in aging studies including cardiovascular investigations [16]. Consequently, F344 was used as comparative strain for an earlier genetic quantitative trait linkage (QTL)-mapping study in a complementary project [17]. Based on their contrasting phenotype we tested the hypothesis that the fetal expression patterns between day 20 of development (E20) and week 14 in adult animals show differences between SHRSP and F344. By using this novel approach we aimed to identify potential candidates that are involved in the development of LVH.

## Methods

### Animals

All rats were obtained from our colonies at the Forschungseinrichtung für experimentelle Medizin (FEM), Charité—Universitätsmedizin, Berlin. SHRSP rats and WKY rats from these colonies were previously described [18,19] and breeder pairs of F344 rats were obtained from Charles River (Charles River Laboratories International, Inc.) to establish a new colony at our facility. The total number of male animals studied were for SHRSP E20 n = 7, week 14 n = 8, for F344 E20 n = 8, week 14 n = 8, and for WKY E20 n = 8, week 14 n = 17. Rats were grouped under a 12:12h light/dark cycle using an automated light switching device and climate-controlled conditions at 22°C. Adult rats at week 14 and maternal animals were fed a normal-salt diet (0.2% NaCl). All animal experiments were approved by the government committee in accordance with national animal protection guidelines (Landesamt für Gesundheit und Soziales (LAGeSo) Berlin, Germany).

At week 14 of age animals were weighed and sacrificed under a ketamine (Ketanest S, Pfizer, Karlsruhe, Germany)/xylazine (Rompun, Bayer, Leverkusen, Germany) anaesthesia (87 mg/kg and 13 mg/kg body weight, respectively).

Fetuses on stage E20 were dissected from the uterus and immediately killed by decapitation. Maternal animals were anesthetized by inhalation of isoflurane (Abbott, Wiesbaden, Germany) and sacrificed by removing the heart.

### Phenotyping

Systolic blood pressure (SBP) was measured at week 14 in conscious animals by the tail-cuff method, which has been previously validated and reported [20]. In brief, two training periods were performed on two separate days. Subsequently, the final blood pressure measurements were recorded on three consecutive days. Due to three sets of two measurements at each session, the individual blood pressure phenotype was based on a maximum of 18 measurements for each rat. A minimum of 12 measurements was required for inclusion in the analysis, which was achieved in all animals.

For determination of cardiac hypertrophy the hearts at week 14 were arrested in diastole by rinsing in 1 M KCl and subsequently carefully blotted dry. The atria were trimmed away and the mass of the whole heart was determined by weighting to the nearest milligram. Removed hearts of E20 animals were rinsed in 1 M KCL and dissected by removing the atria. Hearts were weighted in total to the nearest milligram. In adult hearts LV was separated from septum and right ventricle for RNA isolation. Both ventricles were used for RNA isolation in E20 rats.

### RNA extraction and cDNA synthesis

RNA was isolated from the free wall LV with a trizol assay (TRIzol, Invitrogen Life Technologies) in week 14. Atria free hearts in state E20 were processed with RNeasy Mini Kit (Qiagen, Germany) according to the manufacturer’s instructions. The quality and concentration of the purified total RNA were confirmed by spectroscopy (Nanodrop ND-1000 spectrophotometer; Thermo Fisher Scientific, Waltham, Massachusetts, USA)

First-strand cDNA synthesis was carried out on 2μg of total RNA in a 20μl reaction using the First Strand cDNA Synthesis Kit (Fermentas Life Sciences, St Leon-Rot, Germany) following the manufacturer’s recommendations.

### Microarray analysis

Microarray analysis was performed in SHRSP and F344 samples in individual samples, thus no pooled data were analyzed. For gene expression profiling Illumina RatRef-12 Expression BeadChip with 22523 probes per array was used. Biotinylated, amplified RNA for direct hybridization was generated using the Illumina TotalPrep RNA Amplification Kit (Life Technologies) according to the manufacturer’s instructions. Raw data pre-processing was executed with Genome Studio (Illumina, Version 1.9.0) creating the probe and control profile and the sample information file for further analyses in R (R Core Team. R: A Language and Environment for Statistical Computing. 2013) Quality control and ongoing data processing was performed with package 'lumi' [21]. A log2-transformation and quantile normalization was applied to the data. For differential expression analysis we used the adequately supplied methods from package 'limma' [22]. T-statistics and log-odds of differential expression with empirical Bayes were computed and adjusted with Benjamini & Hochberg. Microarray data is deposited in the Gene Expression Omnibus and available under accession number GSE53512. For functional annotation Gene Ontology Categories [23] were used. Pathways are annotated corresponding to the Kyoto Encyclopedia of Genes and Genomes (KEGG) [24].

### mRNA expression analysis

To quantify mRNA expression of atrial natriuretic peptide (Nppa), potassium voltage-gated channel, Isk-related family, member 1 (Kcne1), EF-hand calcium binding domain 6 (Efcab6), cytoplasmic epoxide hydrolase 2 (Ephx2), caspase recruitment domain family, member 9 (Card9), and defensin beta 1 (Defb1) in heart tissue, we used the quantitative Real time PCR (qPCR) method. Appropriate custom-made primers were designed with the Primer Express software and obtained by TIB Molbiol (Berlin, Germany). Oligonucleotide sequences are presented in S1 Table. The ABI PRISM 7000 SDS instrument and the Power Sybr Green PCR Master Mix (Applied Biosystems, Darmstadt, Germany) were used to perform the assays according to manufacturer’s recommendations. Relative quantification was done using the ΔΔ-ct method. Every sample was measured in triplicate. To normalize our expression data, we used hypoxanthine guanine phosphoribosyl transferase (HPRT) as a house-keeping gene.

### Statistical analysis

All data were summarized as means ± SD and differences between strains were analyzed by student’s T-Test and ANOVA with post-hoc Bonferroni adjustments if not stated otherwise. Differences were considered significant at the level of p < 0.05.

## Results

### Phenotype

**Fig 1:**
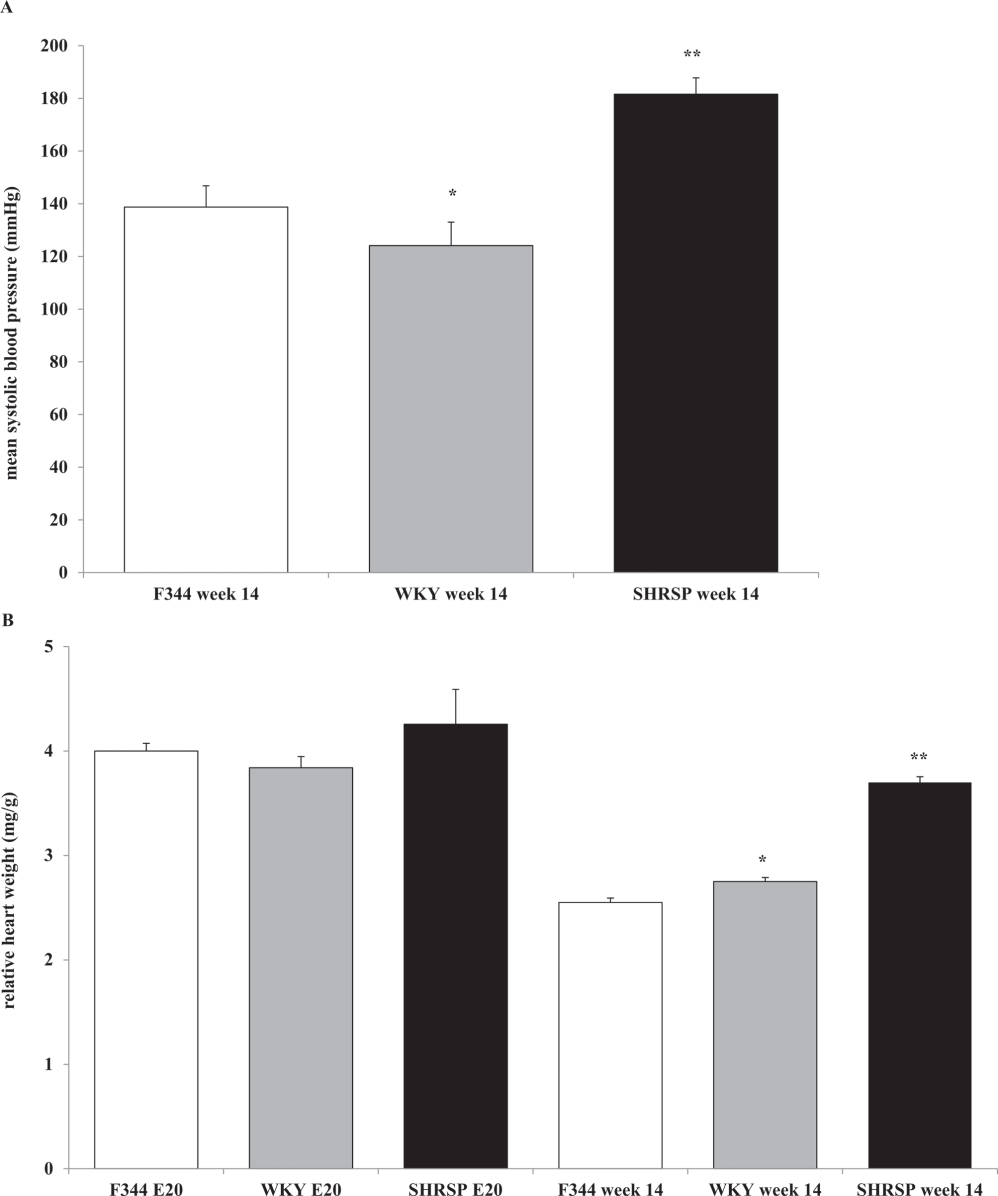
Mean systolic blood pressure and relative heart weight. Comparison of mean systolic blood pressure (panel A) and relative heart weight (panel B) between F344 strain (white bars, E20 n = 8, week 14 n = 8), WKY strain (grey bars, E20 n = 5, week 14 n = 17) and SHRSP (black bars, E20 n = 7, week 14 n = 1) at state E20 and at week 14, respectively. Values are means ± SD, * p < 0.05 vs. F344 at week 14, ** p < 0.05 vs. F344 and WKY at week 14, respectively.

SHRSP showed significantly higher SBP values at week 14 compared to F344 as expected (Fig. 1, panel A). Relative heart weight measurements confirmed significant differences between the three rat strains SHRSP, WKY and F344 at week 14. In state E20 heart weight values were not significantly different (Fig. 1, panel B).

### Regulation of fetal genes

Glucose transporter 4 (Glut4), Pyruvate dehydrogenase kinase 4 (Pdk4) and 2 (Pdk2) as known regulated fetal genes in development showed the expected expression changes during development between E20 and week 14 in SHRSP as well as in F344 in microarray analysis (logFC > |1.5|, p < 0.05). Skeletal alpha actin (Acta1) was regulated in F344 only (logFC = 2.66, p < 0.05) and Malonyl-CoA decarboxylase (Mlycd) showed only a SHRSP specific regulation (logFC = -1.76, p < 0.05). In S2 Table in the supporting information we summarized the expression patterns of known regulated fetal genes in cardiac development.

### Classification of differentially expressed genes

In microarray quality control we demanded intragroup correlation coefficients of expression intensities > 0.95. Samples with lower correlation were removed leaving us with four samples of group SHRSP E20, seven of F344 E20, seven of SHRSP week 14 and eight of F344 week 14. Altogether, 15232 expressed transcripts were detectable below a confident detection threshold of 0.05 and examined for differential gene expression.

**Fig 2:**
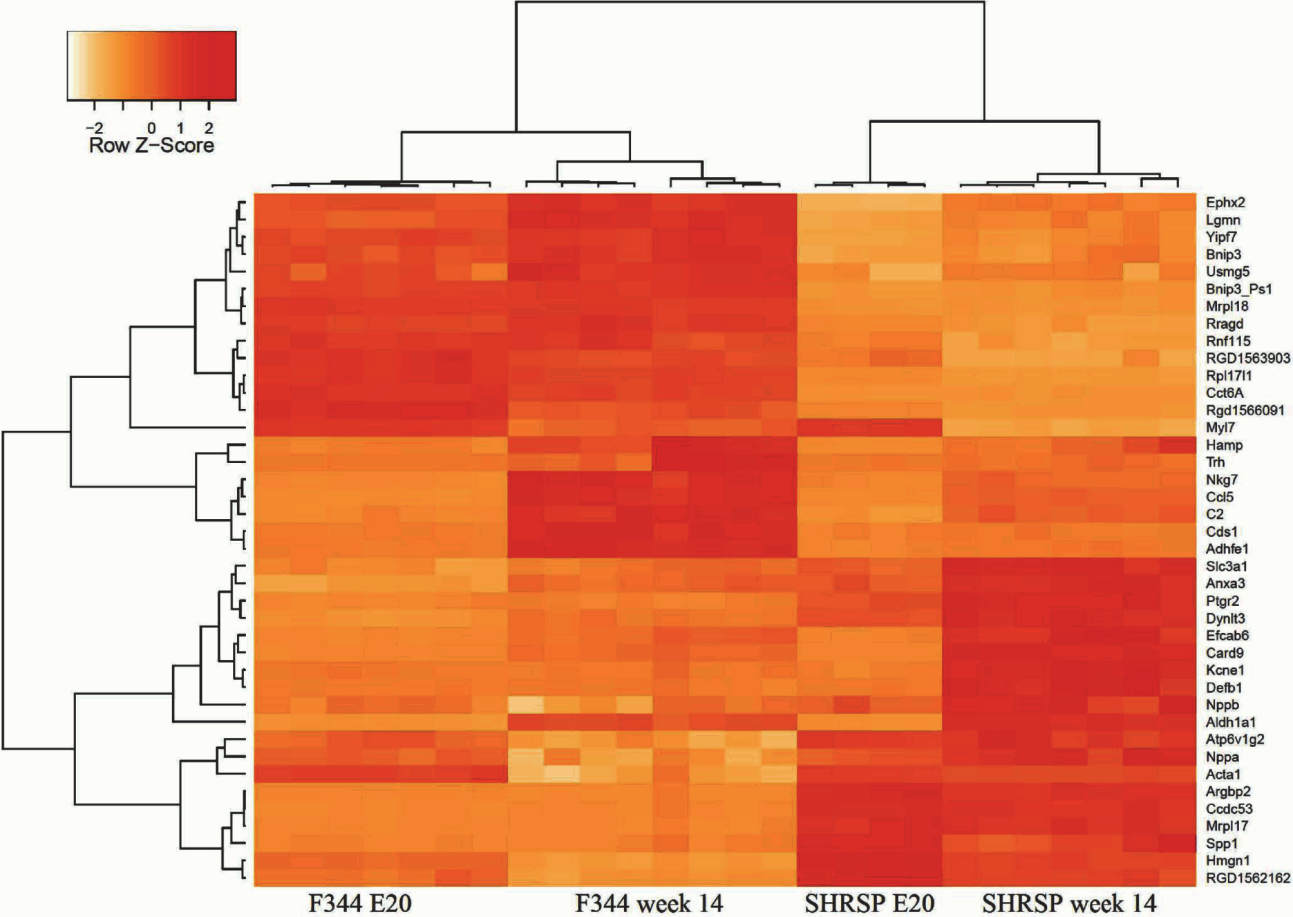
Hierarchical clustering of differentially expressed genes. Hierarchical clustering of differentially expressed genes (log fold change > |1.5| and p < 0.01) between SHRSP and F344 in week 14 over all samples. Samples (columns) and genes (rows) are grouped by their expression profile. Expression values are scaled by Z-Score with zero mean and standard deviation of 1. Values above the mean are shown in red, equivalent to the mean are colored in yellow and below the mean in white. Animal samples of F344 E20 (n = 7), F344 week 14 (n = 8), SHRSP E20 (n = 4) and SHRSP week 14 (n = 7) are in order at the bottom.

For the identification of new potential candidate genes for LVH, we first calculated differentially expressed genes between SHRSP and F344 animals at week 14. Therefore, we applied the 'lmFit' function from package 'limma' of Bioconductor on normalized expression data. Statistics were computed with the 'eBayes' function and p-values were adjusted with False Discovery Rate of Benjamini & Hochberg. For further bioinformatic analysis we considered probes with log fold changes > 1.5 or < -1.5, resulting in 45 transcripts. In SHRSP 24 transcripts showed an up-regulation and 21 transcripts a down-regulation as compared to F344 (log FC > |1.5| and p < 0.05). 40 probes could be annotated with gene name and chromosomal position. A heat map with two-dimensional hierarchical clustering elucidates the expression patterns for the 40 annotated genes over all SHRSP and F344 samples in Fig. 2.

**Fig 3:**
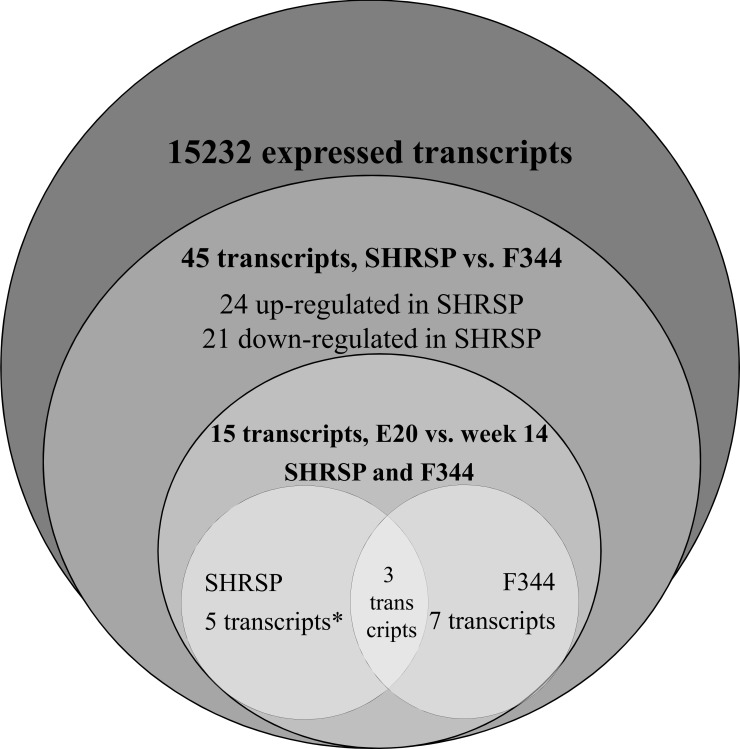
Venn diagram of valid and differentially expressed transcripts. Venn diagram for the candidate identification process to identify genes for further qPCR validation. Significant differential regulation was given with log fold changes > 1.5 or < -1.5 and p < 0.05. First comparison was F344 vs. SHRSP in week 14, second comparison was E20 vs. week 14 in F344 and SHRSP. Validated candidate genes are labelled with asterisk.

To unveil biological functions and cellular processes of these genes we used Gene Ontology terms by which 36 genes could be assigned with at least one term (S1 Fig.) To reveal potential LVH susceptibility genes, we chose a new approach to reduce the earlier detected gene group of 40 specifically expressed probes. Therefore, we analyzed developmental expression patterns in each strain between the time points E20 and week 14 of these genes. Fold change limits were set > 1.5 or < -1.5 as described before. Generally, there were no candidates detected because of a discordant expression with an up-regulation between E20 and week 14 in SHRSP and a down-regulation between E20 and week 14 in F344 or vice versa. In SHRSP 8 genes with a significant differential expression in week 14 compared with F344 showed also a strain specific significant differential expression pattern during development between SHRSP E20 and SHRSP at week 14. In the F344 strain 10 genes with significant differential expression in week 14 compared with SHRSP and during F344 specific development were identified. 3 genes were identified in both strains showing a significant differential expression between E20 and week 14. However, our approach prioritized 5 genes as potential candidates for LVH because they showed only in the hypertensive SHRSP rat a differential expression in comparison with F344 at week 14 and a differential expression pattern during development as well. In contrast the remaining 7 transcripts with F344 specific differential expression were evaluated as less relevant for LVH. Thus, we decided to further explore Kcne1, Efcab6 Ephx2, Card9, and Defb1. Table 1 summarizes the detected genes with fold changes and p-values for the two performed comparisons, i.e. SHRSP versus F344 in week 14 and the strain specific expression pattern between E20 and week 14. Fig. 3 illustrates the candidate gene identification process.

**Table 1:** Fold changes (logFC) and adjusted p-values of candidate genes in SHRSP and F344.

	SHRSP vs. F344 week 14		SHRSP E20 vs. week 14		F344 E20 vs. week 14	
Gene Symbol	logFC	adj. p value	logFC	adj. p-value	logFC	adj. p-value
Acta1	2.36	< 0.001	0.33	0.47	2.67	< 0.001
Adhfe1	-2.08	< 0.001	-0.28	< 0.05	-2.21	< 0.001
Aldh1a1	1.62	< 0.001	-4.72	< 0.001	-3.26	< 0.001
Anxa3	1.6	< 0.001	-1.24	< 0.001	-1.30	< 0.001
Argbp2	4.01	< 0.001	0.55	< 0.001	0.08	0.59
Atp6v1g2	1.6	< 0.001	-0.18	0.4	0.88	< 0.001
Bnip3	-2.42	< 0.001	-0.69	< 0.05	-0.82	< 0.001
Bnip3-Ps1	-4.64	< 0.001	-0.29	0.08	-0.77	< 0.001
C2	-1.52	< 0.001	-1.49	< 0.001	-2.63	< 0.001
Card9*	3.21	< 0.001	-4.36	< 0.001	-1.12	< 0.001
Ccdc53	2.99	< 0.001	0.26	< 0.05	-0.14	0.06
Ccl5	-2.03	< 0.001	-1.57	< 0.001	-3.68	< 0.001
Cct6a	-4.09	< 0.001	0.08	0.66	1.01	< 0.001
Cds1	-1.76	< 0.001	-0.14	0.35	-1.83	< 0.001
Defb1*	1.58	< 0.001	-1.57	< 0.001	-0.03	0.92
Dynlt3	2.03	< 0.001	-1.16	< 0.001	-0.68	< 0.001
Efcab6*	1.67	< 0.001	-2.41	< 0.001	-0.83	< 0.001
Ephx2*	-2.33	< 0.001	-1.64	< 0.001	-1.05	< 0.001
Hamp	-1.76	< 0.001	-1.37	< 0.05	-2.80	< 0.001
Hmgn1	2.55	< 0.001	1.38	< 0.001	1.43	< 0.001
Kcne1*	1.51	< 0.001	-1.66	< 0.001	-0.02	0.92
Lgmn	-1.58	< 0.001	-0.53	< 0.001	-0.86	< 0.001
Mrpl17	2.17	< 0.001	0.20	0.20	0.01	0.95
Mrpl18	-4.12	< 0.001	-0.04	0.82	0.09	0.45
Myl7	-3.76	< 0.001	6.24	< 0.001	2.46	< 0.001
Nkg7	-1.61	< 0.001	-0.64	< 0.001	-2.30	< 0.001
Nppa	2.78	< 0.001	-1.13	< 0.05	1.45	< 0.001
Nppb	1.66	< 0.001	-0.89	< 0.05	0.32	< 0.05
Ptgr2	1.60	< 0.001	-0.82	< 0.001	-0.11	0.16
RGD1562162	1.74	< 0.001	1.00	< 0.001	0.85	< 0.001
RGD1563903	-1.97	< 0.001	1.05	< 0.001	0.62	< 0.001
RGD1566091	-2.03	< 0.001	0.48	< 0.001	1.42	< 0.001
Rnf115	-1.89	< 0.001	0.59	< 0.001	0.16	0.16
Rpl17l1	-3.95	< 0.001	0.61	< 0.001	0.88	< 0.001
Rragd	-1.78	< 0.001	0.29	< 0.05	-0.27	< 0.001
Slc3a1	1.50	< 0.001	-1.16	< 0.001	-0.48	< 0.05
Spp1	1.96	< 0.001	0.80	0.05	-0.05	0.91
Trh	-1.62	< 0.001	-0.27	0.74	-1.85	< 0.001
Usmg5	-1.91	< 0.001	-0.56	0.05	-0.91	< 0.001
Yipf7	-1.63	< 0.001	-0.43	< 0.001	-0.42	< 0.001

Values in the italic written column indicate significantly differentially expressed genes by comparing SHRSP and F344 in week 14 (SHRSP vs. F344). Negative fold changes demonstrate a down-regulation in SHRSP strain. LogFC values in the middle and right columns indicate gene expression by comparing SHRSP E20 and SHRSP in week 14 (E20 vs. week 14, middle column) and F344 E20 and F344 in week 14 (E20 vs. week 14, right column). Negative prefixes indicate a down-regulation in E20. Microarray chip probe ID with annotated gene symbol is indicated if available. Significantly differentially expressed genes with logFC > 1.5 by comparing the strains SHRSP and F344 in week 14 as well as by comparing animals in E20 and week 14 are given in bold. Validated candidate genes are labelled with asterisk.

A mapping of Gene Ontology Terms revealed immune system associated functions for Card9 and Defb1 (e.g. ‘positive regulation of I-kappaB kinase/NF-kappaB cascade’, Gene Ontology Term number (GO): 0005737 and ‘innate immune response’, GO:0045087 for Defb1). Ephx2 could be mapped to ‘catalytic activity’ (GO: 0003824), ‘metabolic process (GO: 0008152)’ or ‘lipid phosphatase activity’ (GO: 0042577) among others. For Efcab6 only one term was found, i.e. ‘calcium ion binding’ (GO: 0005509). Terms like ‘heart contraction’ (GO: 0060047), ‘plasma membrane’ (GO: 0005886) and ‘regulation of heart rate by cardiac conduction’ (GO: 0086091) are assigned to Kcne1. Pathway annotation was only available for two of the five genes; Card9 is involved in the 'NOD-like receptor signalling pathway', whereas Ephx2 is associated with 'Arachidonic acid metabolism', 'Metabolic pathways' and ‘Peroxisome’.

### Verification by qPCR

We performed qPCR analysis for validation of potential candidate genes identified in the microarray analysis. We measured mRNA expression of the known cardiac hypertrophy marker Nppa as positive control. Additionally, we used WKY samples as second control strain to distinguish strain dependent effects.

In week 14 Nppa, Kcne1, Efcab6 and Defb1 showed the highest mRNA levels in SHRSP in comparison with F344 and WKY animals of the same age by qPCR analysis. In this comparison SHRSP animals presented at least a 40% higher expression for these genes compared to both F344 and WKY (p < 0.05, respectively).

In contrast, Ephx2 and Card9 were significant lower (-77%, p < 0.001) or similarly (-6%, p = 0.43) expressed in SHRSP at week 14 in comparison with F344 in qPCR analysis. WKY animals at week 14 showed at least a 30% higher expression for these two genes compared to SHRSP animals (p < 0.01, respectively).

The demonstrated regulation between E20 and week 14 in SHRSP and F344 by microarray analysis could be confirmed by qPCR for Kcne1, Efcab6 and Ephx2. Kcne1, Efcab6 and Ephx2 showed in qPCR analysis an up-regulation of gene expression in SHRSP between E20 and week 14 (p < 0.05, respectively). In contrast, expression value changes during development of these genes were not significant in F344. WKY animals showed up-regulation of Kcne1 as well as of Ephx2 and down-regulation of Efcab6 (p < 0.01, respectively). Defb1 gene expression in E20 was too low for further qPCR analysis.

Card9 was significantly regulated in both of the normotensive control strains (p < 0.05) but not in SHRSP (p > 0.5). Card9 showed discrepant expression values in microarray (significant up-regulation in SHRSP in week 14) and qPCR analysis and was therefore excluded as a candidate, because the detected significant up-regulation in SHRSP week 14 compared to corresponding F344 in microarray analysis was not confirmed by qPCR analysis.

**Fig 4:**
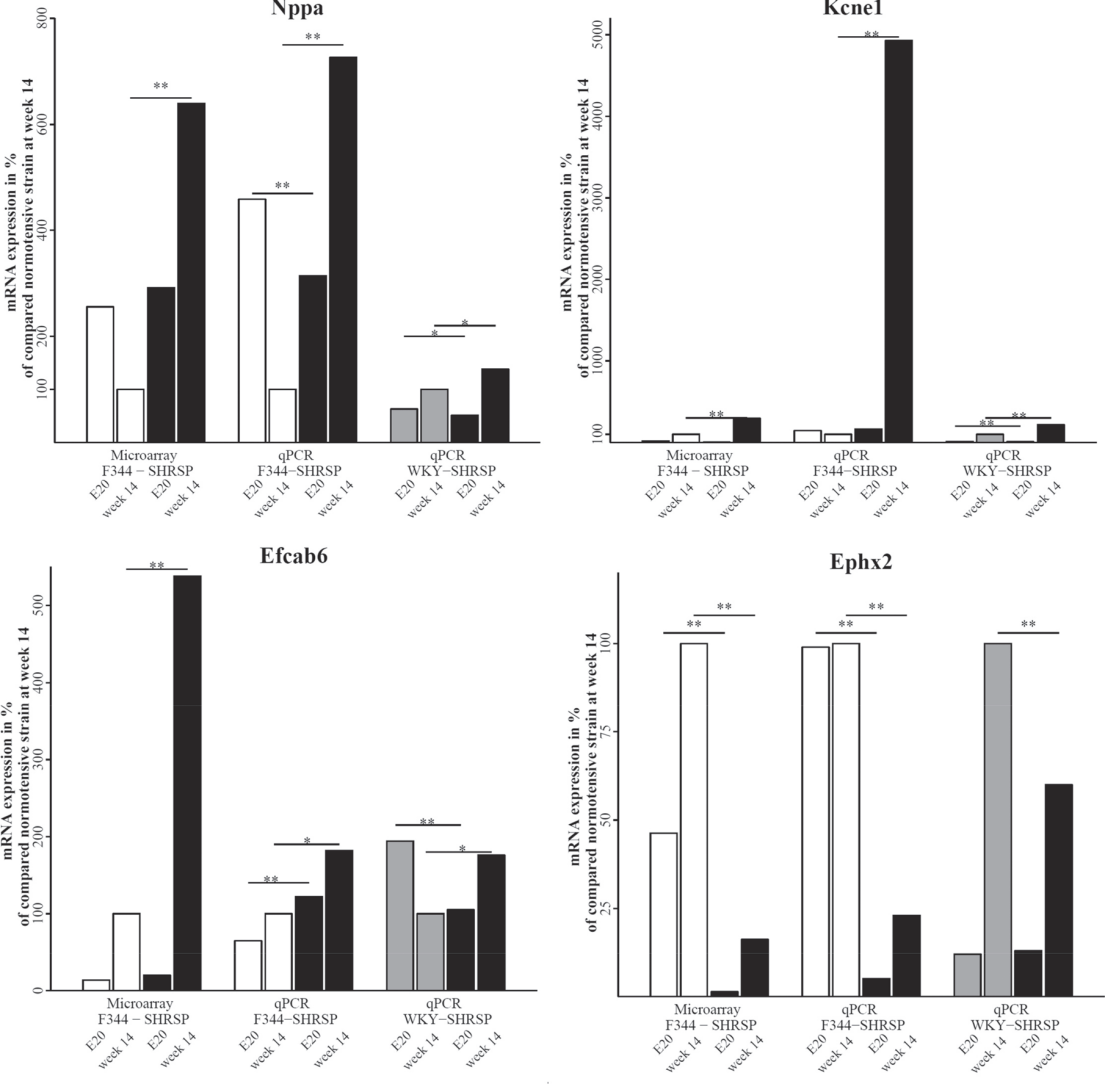
Comparison of gene expression by microarray analysis and qPCR analysis. Comparison of mRNA expression levels of atrial natriuretic peptide (Nppa), potassium voltagegated channel, Isk-related family, member 1 (Kcne1), EF-hand calcium binding domain 6 (Efcab6) and epoxide hydrolase 2 (Ephx2) at day 20 of fetal development (E20) and week 14 of postnatal life between normotensive strains F344 (white bars) or WKY (grey bars) in comparison to SHRSP (black bars). Data for expression analysis by microarray are shown in the left group, for qPCR analysis in the middle and right groups, respectively. Values are given in % of corresponding control strain F344 or WKY at week 14. *, p < 0.05; **, p < 0.01.

Fig. 4 shows mRNA expression levels and regulation between E20 and week 14 by microarray and qPCR analysis.

## Discussion

In this study we used a multi-stage approach to identify genes potentially involved in the development of LVH phenotype by comparing gene expression patterns of two contrasting strains (SHRSP versus F344) and two development stages (fetal E20 versus adult week 14). Our microarray analysis revealed 5 genes as potential candidates for LVH.

As the first step to assess gene expression, we used Illumina BeadArray technology with more than 22k transcripts. 15232 probes were detected as expressed (p < 0.05) and screened for differential expression. Subsequently, we validated our results with qPCR.

We tested Nppa encoding atrial natriuretic peptide as classical marker for LVH as positive control. As expected SHRSP rats at week 14 showed the highest Nppa expression values compared to F344 in microarray analysis (logFC = 2.78, p < 0.001) and in qPCR analysis compared with F344 (+630% compared to F344 at week 14, p < 0.01) and with WKY (+38% of WKY, p < 0.05).

We aimed to identify differences in fetal-adult cardiac expression programs by contrasting two informative rat strains. Our validation experiments do not show similar consistent expression differences between SHRSP and the two normotensive control strains.

Efcab6 and Kcne1 showed the highest gene expression in SHRSP at week 14 and a strong up-regulation during SHRSP development in comparison with both normotensive control strains in qPCR gene expression determination. Efcab6 showed 82% and 76% higher expression in adult SHRSP than in adult F344 and WKY. Efcab6 encodes for EF-hand calcium binding domain 6 and belongs to the large and diverse calcium regulating EF-hand superfamily; the EF-hand calcium binding motif was identified with functional importance in heart diseases in earlier studies [25,26]. Efcab6 is a poorly described transcript and was identified earlier in human as DJBP an oncogene binding partner [27]. A functional role of Efcab6 was not described to our knowledge.

Kcne1 showed in SHRSP and WKY an increase of gene expression during development but a decrease in F344 by comparing qPCR results. Maximum expression values in SHRSP at week 14 were at least about two fold higher than in the two normotensive strains. The influence of Kcne1 in heart failure was previously reported [28]. The strong regulation of Kcne1 resulting in high expression in week 14 may point out to an important adaption process during LVH development in SHRSP.

The further detected candidate gene Ephx2 was previously reported as a promising candidate for cardiac hypertrophy in rodents and patients [29,30]. Elevated Ephx2 expression values were associated with heart failure in rats [30]. However, in cardiac tissue of heart failure patients Ephx2 expression was found to be down-regulated [30]. Similarly, in our work Ephx2 gene expression was at least about 40% lower in SHRSP than in the control strains F344 or WKY, respectively. Furthermore, earlier studies identified Ephx2 as interesting drug target in heart failure [31,32]. Its influence on epoxyeicosatrienoic acids homeostasis has been suggested to modulate cardiac function but the mechanism is still not completely understood and needs to be clarified in further studies [30,31]. The latter is important, since our study does per se not allow differentiating whether the identified candidates are primarily, i.e. causative, involved in the development of LVH or whether they are related to cardiac adaption processes resulting in larger heart size in SHRSP [33]. Furthermore, differentially expressed genes could be still a general sign for genomic strain differences without relevance for cardiac hypertrophy development.

The basis of our study is the phenotypical difference between F344 and SHRSP in blood pressure and heart weight. In this regard it is important to consider the well-known influence of elevated blood pressure on gene regulation [34]. Thus, differences in cardiac gene expression in adult animals might occur as secondary changes in response to the elevated blood pressure. A follow-up study is therefore needed where the effect of blood pressure on the target genes, e.g. by reducing blood pressure in SHRSP to the same level as F344, could be investigated.

As a limitation of our study gene expression was measured in total LV tissue in adult and in whole heart samples of fetal E20 animals. Thus, we did not differentiate between right and LV tissue in E20 animals, although it should not be dismissed that the hemodynamic exposure of cardiac ventricles in E20 animals is per se quite different from the adult animals.

In light of the fact that both cardiomyocytes and cardiac fibroblasts play an important role in LVH development and cardiac remodelling [35–37] our data do not allow distinguishing expression profiles between the two cell types. Furthermore, although the left ventricle is the primary and main target for hypertensive end organ damage, differences in gene expression profiles between cardiac tissues from the ventricle and atria have been reported earlier in addition to differences between cardiac myocytes and cardiac fibroblast [38–41].

We used only Hprt as house-keeping gene in qPCR analyses. Although Hprt is an established house-keeping gene in cardiac gene expression studies [42,43] and our data demonstrated only a low variability of Hprt expression between the analyzed strains and age groups, the use of additional house-keeping genes could have increased the validity of our analysis [44].

A further limitation is related to the fact that we measured heart weight and gene expression on two time points only, i.e. state E20 and week 14 as development time points of late gestation age and young adulthood, respectively. In this regard it is of interest, that a high cardiac metabolic gene expression including mitochondrial biogenesis genes and glucose transporter Glut-1 in rats at gestation age of E20 with a subsequent decrease has been reported [45]. The chosen time distance might also be an explanation for the observation that a significant differential expression of the known markers for cardiac development like Myh6 and Myh7 were missed in our analysis. Furthermore we could detect strain dependent difference in developmental regulation for Acta1 and Mlycd. The expression of these fetal genes may therefore be influenced by factors including cardiac mechanical influences that differ between the two analyzed rat strains [46]. Nevertheless we could verify representative markers for transcriptional changes in cardiac development in both strains [11].

The regulation of Nppa and F344 specific genes are discussed in the S1 Discussion Appendix.

To our knowledge, in this work we report a new approach to identify candidate genes for cardiac hypertrophy by analyzing both gene expression differences between strains with contrasting cardiac phenotype in combination with a comparison of cardiac fetal-adult gene expression patterns in the same strains. We thus identified Efcab6 as new potential candidate gene for the LVH phenotype and confirmed earlier published candidate genes. The functional relevance of the identified candidate genes for LVH needs to be tested in future studies, to exclude the possibility that their observed expression difference is solely related to a genetic inter-strain difference without functional relevance for LVH. Further validation and functional experiments are necessary to confirm these targets and new mechanisms related to cardiac hypertrophy.

## Supporting Information

S1 Fig Gene ontology (GO)-Term annotation. Gene ontology (GO)-Term annotation for differentially expressed genes in SHRSP at week 14 in comparison with F344. Panel A represents GO-Terms for ‘biological process’ in SHRSP specific up-regulated genes, Panel B shows SHRSP specific down-regulated genes. Panels C and D demonstrate annotated genes for class ‘cellular component’ in corresponding order as described above. Panel E and F represent annotated genes of ‘molecular function’ (order as above). Categories with more than two genes in one group are shown. The numbers indicate the number of associated genes per term.
(TIF)

S1 Table Oligonucleotide sequences of used primer for qPCR validation. Gene symbol and NCBI Gene ID of candidate genes is indicated.
(DOCX)

S2 Table Fold changes (logFC) and adjusted p-values of known cardiac development marker in SHRSP and F344 by microarray. Values in the middle and right columns indicate gene expression by comparing SHRSP E20 and SHRSP in week 14 (E20 vs. week 14, middle column) and F344 E20 and F344 in week 14 (E20 vs. week 14, right column). Microarray chip probe ID with annotated gene symbol is indicated. Significantly differentially expressed genes with logFC > 1.5 gene expression in E20 and week 14 are given in bold. Not significant differential expression is indicated with n.s.
(DOCX)

S1 Discussion Appendix A down-regulation of Nppa expression after birth in rat hearts has been previously described [47] and F344 exhibited lower Nppa expression in adult compared to E20 animals by qPCR analysis. In contrast, LV Nppa expression increased in adult animals at week 14 compared to E20 animals in both SHRSP and WKY. This finding could be related to the smaller cardiac growth and/or adaptive cardiac response during aging in the F344 strain compared to both SHRSP and WKY. In addition, expression values of Nppa were significantly higher in E20 animals of F344 as compared to SHRSP (+ 20%, p < 0.01) which could point to a possible cardiac developmental mismatch between the two strains resulting in different cardiac expression patterns. Genes that showed a specific expression pattern with up-regulation only in F344, e.g. Adhfe1, were not further examined in the presented work, because we prioritized our analysis on up-regulated genes in SHRSP. Thus, potential candidate genes with LVH preventing function were not validated and could be studied in the further.
(DOC)
