# Supplementary figures and images for: Fetal-Adult Cardiac Transcriptome Analysis in Rats with Contrasting Left Ventricular Mass Reveals New Candidates for Cardiac Hypertrophy
Source: PLoS One. 2015 Feb 3;10(2):e0116807. doi: 10.1371/journal.pone.0116807 (PMC4315412; doi:10.1371/journal.pone.0116807)

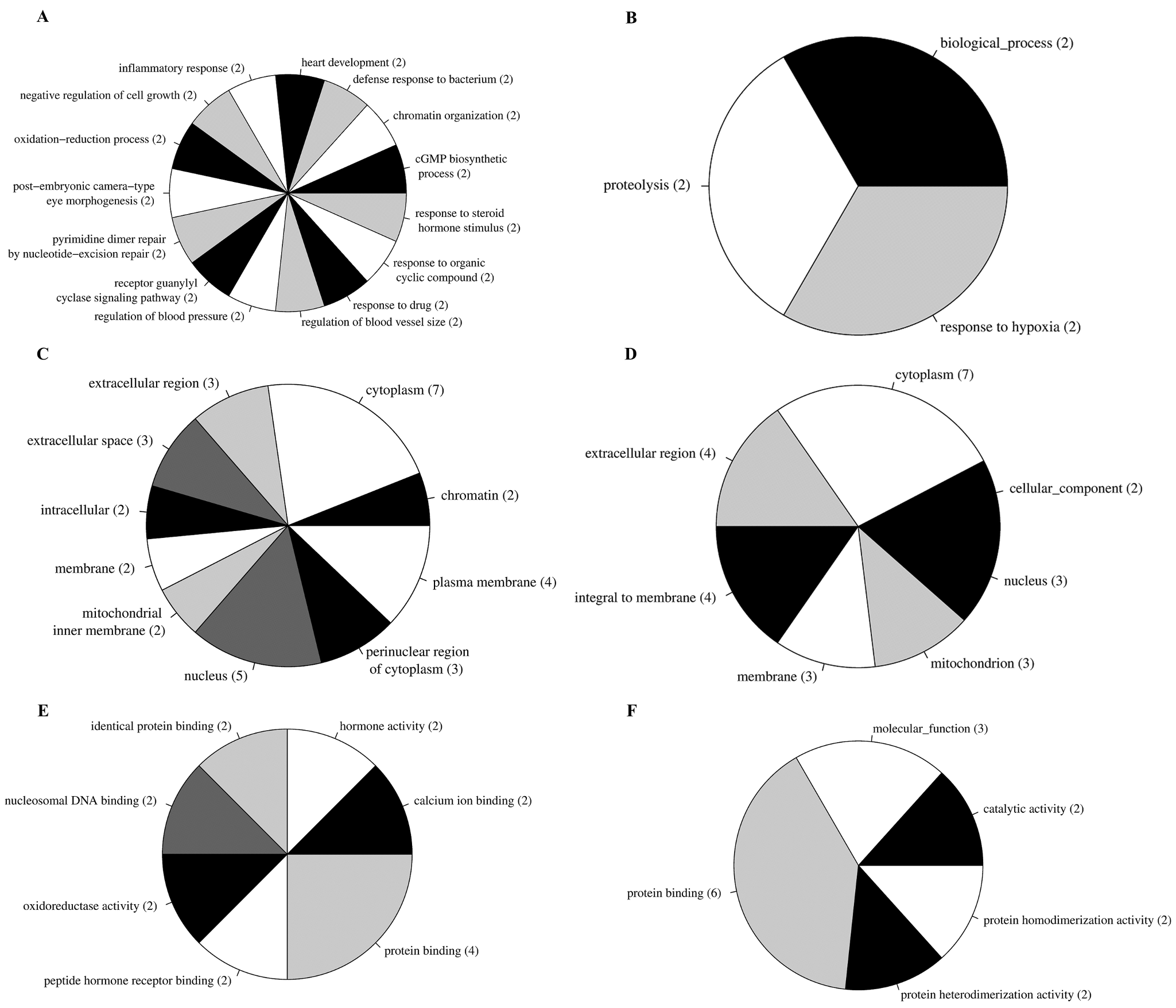

Supplement: S1 Fig — Gene ontology (GO)-Term annotation for differentially expressed genes in SHRSP at week 14 in comparison with F344. Panel A represents GO-Terms for ‘biological process’ in SHRSP specific up-regulated genes, Panel B shows SHRSP specific down-regulated genes. Panels C and D demonstrate annotated genes for class ‘cellular component’ in corresponding order as described above. Panel E and F represent annotated genes of ‘molecular function’ (order as above). Categories with more than two genes in one group are shown. The numbers indicate the number of associated genes per term. (TIF) [file pone.0116807.s001.tif]
