# Supplementary material for: Fetal-Adult Cardiac Transcriptome Analysis in Rats with Contrasting Left Ventricular Mass Reveals New Candidates for Cardiac Hypertrophy
Source: PLoS One. 2015 Feb 3;10(2):e0116807. doi: 10.1371/journal.pone.0116807 (PMC4315412; doi:10.1371/journal.pone.0116807)
Supplement: S1 Table — Gene symbol and NCBI Gene ID of candidate genes is indicated. (DOCX) [file pone.0116807.s002.docx]

**Table S1**

| **Gene Symbol** | **NCBI Gene ID** | **Sense primer** | **Antisense primer** |
| --- | --- | --- | --- |
| Card9 | 64171 | CCTATCTGCGCCAGTGCAA | AGACTCTCAAGAAAGGCCACGT |
| Ephx2 | 65030 | GCAGCTGCTGCTCTCAAAAA | CCTGACAGGACTCTATGAGGAAGTC |
| Defb1 | 83687 | AGCTGGGTGCTGGCATTC | AGTTGGGCTTATCTGGTTTACATGT |
| Efcab6 | 315179 | ATGAATGACTGCCAATATGCAATG | TTTAGAGGTTGGGCGTGACTG |
| Kcne1 | 25471 | CTCGCTGTGGCAGGAAACA | GCCCAGGGTGAAGAAGCC |
| Nppa | 24602 | CTGATGGATTTCAAGAACCTGCTA | CACCTCCATCTCTCTGAGACGG |
| Hprt | 24465 | CTCATGGACTGATTATGGACAGGACT | TCCAGCAGGTCAGCAAAGAAC |
