# Supplementary material for: Fetal-Adult Cardiac Transcriptome Analysis in Rats with Contrasting Left Ventricular Mass Reveals New Candidates for Cardiac Hypertrophy
Source: PLoS One. 2015 Feb 3;10(2):e0116807. doi: 10.1371/journal.pone.0116807 (PMC4315412; doi:10.1371/journal.pone.0116807)
Supplement: S2 Table — Values in the middle and right columns indicate gene expression by comparing SHRSP E20 and SHRSP in week 14 (E20 vs. week 14, middle column) and F344 E20 and F344 in week 14 (E20 vs. week 14, right column). Microarray chip probe ID with annotated gene symbol is indicated. Significantly differentially expressed genes with logFC > 1.5 gene expression in E20 and week 14 are given in bold. Not significant differential expression is indicated with n.s. (DOCX) [file pone.0116807.s003.docx]

**Table S2**

| **Gene symbol** | **SHRSP E20 vs. week 14** | | **F344 E20 vs. week 14** | |
| --- | --- | --- | --- | --- |
|  | **logFC** | **adj. p-value** | **logFC** | **adj. p-value** |
| Myh6 | -0.56 | < 0.05 | -0.55 | < 0.05 |
| Myh7 | -0.07 | n.s. | 0.18 | n.s. |
| Actc1 | -0.39 | < 0.05 | -0.12 | n.s. |
| **Acta1** | 0.33 | n.s. | **2.66** | **< 0.05** |
| **Slc2a4** | **-2.02** | **< 0.05** | **-2.31** | **< 0.05** |
| Cpt1b | -1.36 | < 0.05 | -1.49 | < 0.05 |
| Ckm | -0.07 | n.s. | -0.3 | < 0.05 |
| Ppara | -0.1 | n.s. | -0.008 | n.s. |
| **Pdk2** | **-1.84** | **< 0.05** | **-1.73** | **< 0.05** |
| **Pdk4** | **-3.24** | **< 0.05** | **-3.23** | **< 0.05** |
| **Mlycd** | **-1.76** | **< 0.05** | -1.48 | < 0.05 |
