# Supplementary material for: Fetal-Adult Cardiac Transcriptome Analysis in Rats with Contrasting Left Ventricular Mass Reveals New Candidates for Cardiac Hypertrophy
Source: PLoS One. 2015 Feb 3;10(2):e0116807. doi: 10.1371/journal.pone.0116807 (PMC4315412; doi:10.1371/journal.pone.0116807)
Supplement: S1 Discussion Appendix — In contrast, LV Nppa expression increased in adult animals at week 14 compared to E20 animals in both SHRSP and WKY. This finding could be related to the smaller cardiac growth and/or adaptive cardiac response during aging in the F344 strain compared to both SHRSP and WKY. In addition, expression values of Nppa were significantly higher in E20 animals of F344 as compared to SHRSP (+ 20%, p < 0.01) which could point to a possible cardiac developmental mismatch between the two strains resulting in different cardiac expression patterns. Genes that showed a specific expression pattern with up-regulation only in F344, e.g. Adhfe1, were not further examined in the presented work, because we prioritized our analysis on up-regulated genes in SHRSP. Thus, potential candidate genes with LVH preventing function were not validated and could be studied in the further. (DOC) [file pone.0116807.s004.doc]

**Discussion appendix S1**

A down-regulation of Nppa expression after birth in rat hearts has been previously described [1] and F344 exhibited lower Nppa expression in adult compared to E20 animals by qPCR analysis. In contrast, LV Nppa expression increased in adult animals at week 14 compared to E20 animals in both SHRSP and WKY. This finding could be related to the smaller cardiac growth and/or adaptive cardiac response during aging in the F344 strain compared to both SHRSP and WKY. In addition, expression values of Nppa were significantly higher in E20 animals of F344 as compared to SHRSP (+ 20%, p < 0.01) which could point to a possible cardiac developmental mismatch between the two strains resulting in different cardiac expression patterns.

Genes that showed a specific expression pattern with up-regulation only in F344, e.g. Adhfe1, were not further examined in the presented work, because we prioritized our analysis on up-regulated genes in SHRSP. Thus, potential candidate genes with LVH preventing function were not validated and could be studied in the further.

Reference List

1. Houweling AC, van Borren MM, Moorman AF, Christoffels VM (2005) Expression and regulation of the atrial natriuretic factor encoding gene Nppa during development and disease. Cardiovasc Res 67: 583-593. S0008-6363(05)00289-0 [pii];10.1016/j.cardiores.2005.06.013 [doi].
